# Targeted Drug Designing for Treating Masticatory Myofascial Pain Dysfunction Syndrome: An In Silico Simulation Study

**DOI:** 10.7759/cureus.51661

**Published:** 2024-01-04

**Authors:** Ramya Suresh, Ramya Ramadoss, Mukesh Doble, Karthikeyan Ramalingam, Sandhya Sundar, Suganya Panneer Selvam

**Affiliations:** 1 Oral Biology, Saveetha Dental College and Hospitals, Saveetha Institute of Medical and Technical Sciences, Saveetha University, Chennai, IND; 2 Oral Pathology and Microbiology, Saveetha Dental College and Hospitals, Saveetha Institute of Medical and Technical Sciences, Saveetha University, Chennai, IND; 3 Conservative Dentistry and Endodontics, Saveetha Dental College and Hospitals, Saveetha Institute of Medical and Technical Sciences, Saveetha University, Chennai, IND; 4 Oral Pathology and Microbiology, Saveetha Dental College and Hospital, Saveetha Institute of Medical and Technical sciences, Saveetha University, Chennai, IND; 5 Oral Pathology and Microbiology, Saveetha Dental College and Hospital, Saveetha Institute of Medical and Technical Sciences, Saveetha University, Chennai, IND

**Keywords:** docking, computer-aided drug design, stress, bruxism, ligand, myosin, potential drugs, drug discovery, myofascial pain, molecular docking

## Abstract

Background

Masticatory Myofascial Pain Dysfunction Syndrome (MMPDS) is a musculoligamentous disorder that shares similarities with temporomandibular joint pain and odontogenic pain. It manifests as dull or aching pain in masticatory muscles, influenced by jaw movement. Computer-aided drug design (CADD) encompasses various theoretical and computational approaches used in modern drug discovery. Molecular docking is a prominent method in CADD that facilitates the understanding of drug-bimolecular interactions for rational drug design, mechanistic studies & the formation of stable complexes with increased specificity and potential efficacy. The docking technique provides valuable insights into binding energy, free energy, and complex stability predictions.

Aim

The aim of this study was to use the docking technique for myosin inhibitors.

Materials and methods

Four inhibitors of myosin were chosen from the literature. These compound structures were retrieved from the Zinc15 database. Myosin protein was chosen as the target and was optimized using the RCSB Protein Data Bank. After pharmacophore modeling, 20 novel compounds were found and the SwissDock was used to dock them with the target protein. We compared the binding energies of the newly discovered compounds to those of the previously published molecules with the target.

Results

The results indicated that among the 20 molecules ZINC035924607 and ZINC5110352 exhibited the highest binding energy and displayed superior properties compared to the other molecules.

Conclusion

The study concluded that ZINC035924607 and ZINC5110352 exhibited greater binding affinity than the reported inhibitors of myosin. Therefore, these two molecules can be used as a potential and promising lead for the treatment of MMPDS and could be employed in targeted drug therapy.

## Introduction

Masticatory myofascial pain dysfunction syndrome (MMPDS) is a condition that affects the muscles and ligaments involved in jaw movement and chewing. It is characterized by chronic dull/aching pain in the jaw, face, and neck muscles. Pain is often associated with temporomandibular disorders which have the highest prevalence of about 19% in the orofacial region next to odontological pain and are clinically expressed as masticatory myofascial pain. The exact cause of MMPDS is not fully understood but it is believed to result from the presence of trigger points in the masticatory muscles [1]. Trigger points are hyper-irritable areas within a muscle that are sensitive to touch and can cause referred pain to other areas. Common factors such as stress, bruxism, poor posture, trauma and muscle overuse can contribute to the development of these trigger points. The diagnosis of MMPDS is typically based on the patient's symptoms and a thorough clinical examination is required. Treatment approaches for MMPDS aim to alleviate pain, improve muscle function and address any underlying contributing factors present. This can involve a combination of therapies, including pharmacotherapy, physical therapy, stress management techniques and the use of oral appliances to reduce bruxism. In addition to conventional treatment treatments, recent research has explored the use of computer-aided drug design (CADD) in identifying potential therapeutic molecules for MMPDS. CADD involves the use of computational methods to model and predict the interactions between drugs and their target molecules. By employing molecular docking techniques, we can assess various compounds' binding affinity and efficacy in targeting specific proteins or receptors involved in MMPDS. Molecular docking is a strong and effective tool for in silico screening. It is becoming more and more important in the process of designing sensible medications. It is a computational process that finds an appropriate ligand that suits the protein's binding site both energetically and geometrically [1,2].

In silico drug design can be utilized to find new leads or hits against specific biologically active macromolecules by using theoretical and computational methods. To discover, establish, and assess pharmaceuticals and other physiologically active compounds, CADD techniques like virtual screening, pharmacophore modeling, molecular docking, and dynamic simulation are currently commonly employed. While an in silico molecular docking approach may easily assess the binding affinities of a large-scale compound with a target macromolecule, a structure and ligand-based pharmacophore model in CADD can find similar active compounds against a particular target protein [2]. A compound's biological activity can be assessed when it binds to a particular macromolecule and causes a specific reaction. The molecular docking approach simplifies and expedites the estimation of a compound's binding capacity, which was formerly a time-consuming and costly process that needed a large-scale in vitro and in vivo trial in conventional drug development. Properties of pharmacokinetics and pharmacology, such as absorption, distribution, metabolism, excretion (ADME), and toxicity, can be anticipated using a CADD approach [2]. This study aimed to demonstrate the CADD approach to identify a potent molecule for targeting myosin protein and to assess the efficacy of new structures in treating masticatory myofascial pain syndrome.

## Materials and methods

**Figure 1:**
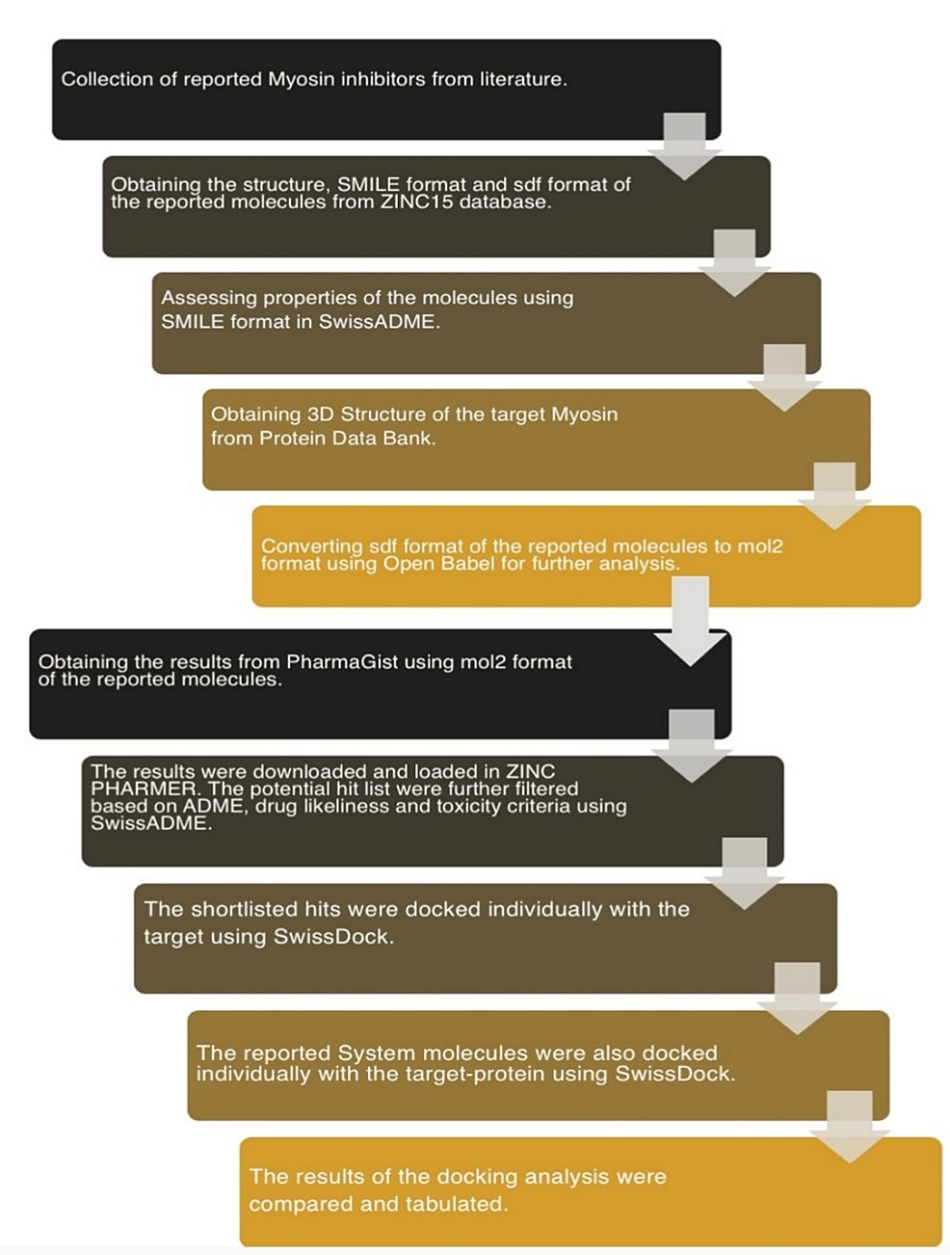
Representation of study workflow. SMILE-Simplified Molecular Input Line Entry System; ADME- Absorption, Distribution, Metabolism and Excretion; SDF- Structure Data file; MOL2 - Molecule

The study was conducted following the acquisition of approval from the Scientific Review Board (SRB/SDC/FACULTY/22/OBIO/113) of the Department of Oral Biology, Saveetha Dental College and Hospitals. The present study targeted the protein myosin. Four potential inhibitors were identified through the analysis of databases such as Google Scholar and PubMed: (1) pyridostigmine, (2) rivastigmine, (3) benztropine, and (4) butanedione. Each of these inhibitors possesses unique clinical applications and functions. The protein utilized in the docking investigation was acquired via the process of homology modeling. Therefore, using the chemical structures of these five molecules as a template for the novel compounds, the present study aims to evaluate the binding affinity of these medications with the targeted myosin protein in the form of binding patterns. Using the workflow abstract shown in Figure 1, the following procedures are followed to create new pharmacophores and evaluate how these compounds interact with the target [3].

Docking procedure

**Figure 2:**
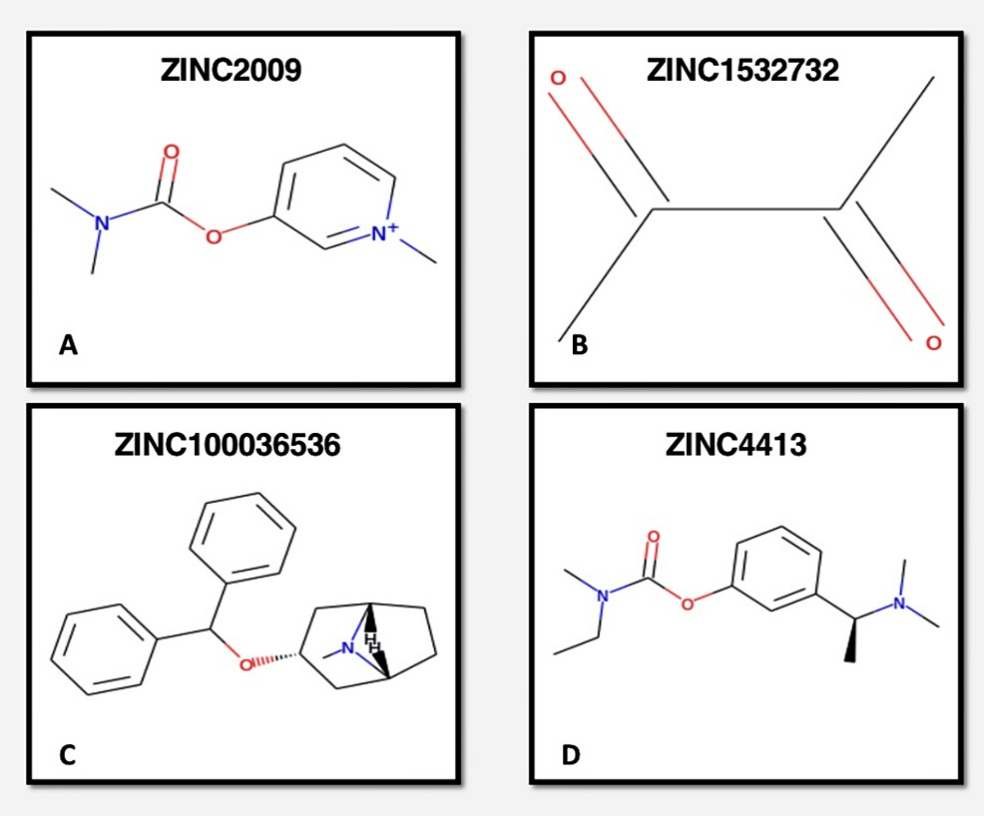
Structures of selected myosin inhibitors from zinc15 database: a) pyridostigmine; b) butanedione; c) benztropine; d) rivastigmine

ZINC15 is a freely accessible chemical database containing dock-ready compounds for purchase [4]. The ZINC15 database was queried for the structures of the chosen molecules. For additional evaluation in SwissADME, the Structure Data File (SDF) and SMILES (Simplified Molecular Input Line Entry Specification) formats of the molecules were acquired. The database was utilized to pick four myosin inhibitors with various structural characteristics, namely pyridostigmine, rivastigmine, benztropine, and butanedione (Figure 2). These compounds were chosen based on their documented significant biological action, as reported in the literature. The chemical structure drawing application, ChemOffice 2004 (CambridgeSoft, Cambridge, USA), was utilized to depict the 2D structures of the myosin inhibitors. The conformational energy of inhibitors was lowered through the utilization of UCSF Chimera (University of California San Francisco, San Francisco, USA). Subsequently, the reduced structures underwent docking studies.

The main goal of molecular docking is to achieve a ligand-receptor complex with an optimized conformation and lower binding free energy. Numerous ligands' binding affinities are anticipated using molecular docking techniques. In this study, we investigated the potential relationship between the experimental bioactivities of the investigated inhibitors and their docking scores. To acquire realistic results, all docking experiments were executed with the default parameters.

**Figure 3:**
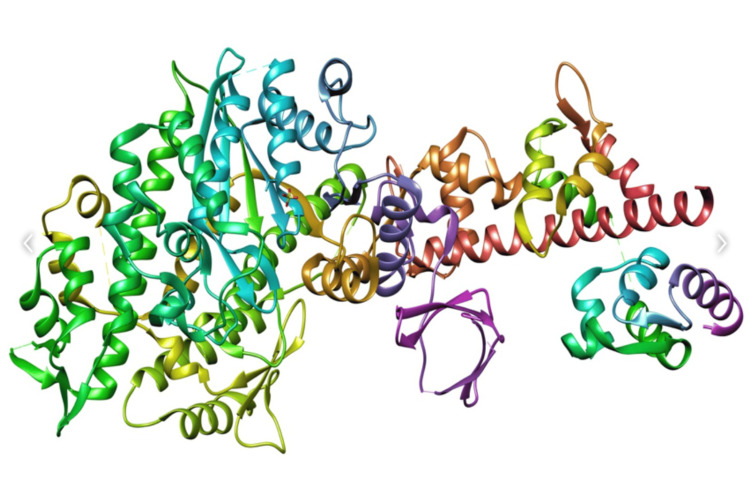
Protein structure of the target myosin Crystal structure of myosin protein with essential light chain retrieved from the Research Collaboratory for Structural Bioinformatics (RCSB) Protein Data Bank (PDB).

Docking in conjunction with a scoring function can be utilized to evaluate large databases to identify in silico potent drug candidates capable of targeting the molecule of interest. Research Collaboratory for Structural Bioinformatics (RCSB) Protein Data Bank (PDB) is an exceptional resource that offers experimentally determined three-dimensional structures of proteins, nucleic acids, and other macromolecules. The target protein myosin's sequence was obtained from the PDB, which is shown in Figure 3.

Selection of Ligands for Inhibitor Design

**Figure 4:**
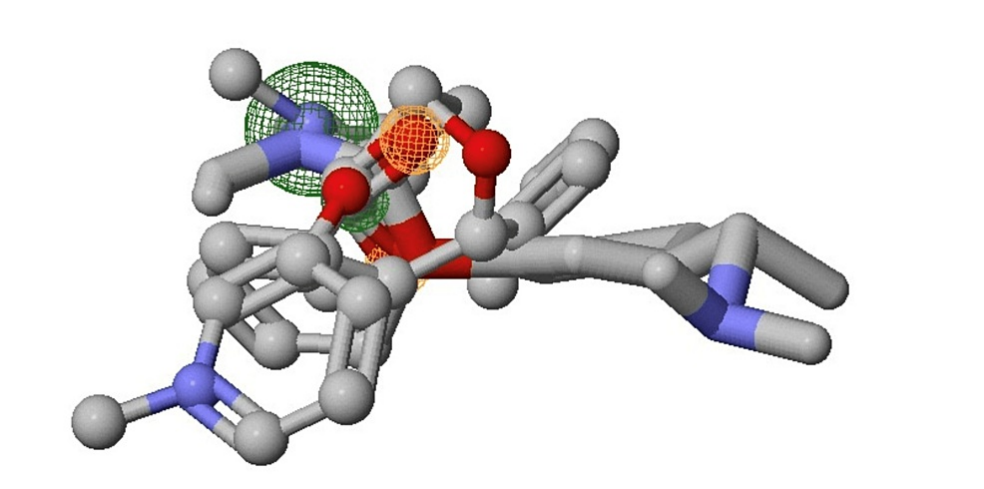
Ligand binding sites. Three-dimensional pharmacophore ligand binding site retrieved from Zinc Pharmer

The four potent drugs were selected based on their inhibitory property against previous studies. The following are the potent drugs selected against the target myosin protein: (1) pyridostigmine, (2) rivastigmine, (3) benztropine, and (4) butanedione. Three-dimensional ligand-binding sites are shown in Figure 4; the two-dimensional chemical structures of the compounds were obtained from the ZINC15 database in structured data format (SDF). The SDF format was then translated to PDB format using PyMOL software (Schrödinger, Inc., New York, USA) for subsequent analysis.

3D Protein Structure Modeling

SwissADME (Swiss Institute of Bioinformatics, Lausanne, Switzerland) is a free software tool that evaluates the chosen compound's pharmacokinetic characteristics, drug-likeness, and medicinal chemistry friendliness to facilitate drug discovery. In SwissADME, the SMILES format from Zinc15 of each molecule was utilized to evaluate features such as Log P, topological polar surface area (TPSA), drug likeliness rules like Lipinski (Rule no. 1), Ghose (Rule no. 2), Veber (Rule no. 3), Egan (Rule no. 4), and Muegge (Rule no. 5). The SWISS-MODEL online server (University of Basel, Basel, Switzerland) was utilized to estimate the 3D structure of the target protein by the application of the homology modeling method. The validation of the 3D protein structures was subsequently performed using the Ramachandran plot [5].

Drug-Likeness Test - SwissADME Software Based on the Lipinski Criteria

To conduct docking studies, it is necessary for the four potent medications to successfully pass a drug-likeness test, which is employed to assess the suitability of a given chemical for usage as a pharmaceutical agent. The assessment of drug-likeness for all compounds was conducted utilizing the SwissADME drug discovery program [5]. The program in question is a rapid, precise, and user-friendly tool for predicting absorption, distribution, metabolism, and excretion processes. Additionally, it can forecast pharmaceutically significant characteristics of organic compounds either alone or in groups. The software assesses compounds using the Lipinski rule, which outlines the criteria for an effective oral drug. According to this rule, the molecular weight of the compound should fall within the range of 131 to 726 Da, the log P value should be less than 5 and the number of hydrogen bond donors should be between 0 and 6, the number of hydrogen bond acceptors should be between 2 and 20, and the number of rotatable bonds should be exceeded till number 5. In addition to conducting drug-likeness assessments, the molecular volume of the compounds must fall within the range of 500 to 2000 [6].

Docking protein and ligand molecules

Pharmacophore Modeling

The purpose of pharmacophore modeling is to generate pharmacophore models by utilizing structural data about the compounds or molecules being investigated. To generate innovative compounds from the established myosin inhibitors, this research employed ZINCPharmer (University of Pittsburg, Pittsburg, USA) and SwissDock (Swiss Institute of Bioinformatics, Lausanne, Switzerland) to identify biologically significant compounds associated with the five existing inhibitors and calculate their binding energies to the target, respectively.

PharmaGist

PharmaGist (Tel Aviv University, Tel Aviv, Israel) is a free web server utilized for detecting pharmacophores, which facilitate the interaction of a molecule with a particular target. Using Open Babel, the molecules from the ZINC15 database were converted from the SDF format to the mol2 (molecule structure format) format before being uploaded to Pharma Gist. The obtained results were subsequently utilized for further analysis.

ZINCPharmer

ZINCPharmer, a freely available pharmacophore search software, autonomously detects biologically significant compounds. The PharmaGist results were imported into ZINCPharmer, where pharmacophores were potentially identified. A subset of 20 molecules, identified by their highest score, was chosen for additional analysis (Table 1). Using the PharmaGist software, pharmacophore modeling is done for four molecules and validated. Twenty hit potential molecules were selected and docked with the target myosin protein using the SwissDOCK software.

**Table 1:** New pharmacophores with their properties Log P- Partition coefficient; TPSA- Topological Polar Surface Area Drug likeliness Rules: Lipinski (Rule 1), Ghose (Rule 2), Veber (Rule 3), Egan (Rule 4), and Muegge (Rule 5). Binding energy- Sum of all the intermolecular interactions present between ligand & target.

S. No	Name	Rule1	Rule2	Rule3	Rule4	Rule5	LogP	Bioavailability	TPSA	Binding Energy (kcal/mol)
1	ZINC03374810	Yes	Yes	Yes	Yes	Yes	3.34	0.55	98.52	-8.55
2	ZINC035924607	Yes	Yes	Yes	Yes	Yes	4.34	0.55	87.72	-9.51
3	ZINC69713715	Yes	Yes	Yes	Yes	Yes	3.14	0.55	76.83	-8.13
4	ZINC35924594	Yes	Yes	Yes	Yes	Yes	4.47	0.55	87.72	-8.68
5	ZINC76718498	Yes	Yes	Yes	Yes	Yes	3.92	0.55	85.64	-8.65
6	ZINC527605	Yes	Yes	Yes	Yes	Yes	2.68	0.55	112.45	-8.21
7	ZINC78824973	Yes	Yes	Yes	Yes	Yes	2.57	0.55	94.34	-8.34
8	ZINC36996340	Yes	Yes	Yes	Yes	Yes	3.21	0.55	29.36	-7.87
9	ZINC1076772	Yes	Yes	Yes	Yes	Yes	3.45	0.55	44.12	-7.89
10	ZINC12308764	Yes	Yes	Yes	Yes	Yes	3.29	0.55	62.83	-8.29
11	ZINC24782747	Yes	Yes	Yes	Yes	Yes	3.3	0.55	72.94	-8.22
12	ZINC55196640	Yes	Yes	Yes	Yes	Yes	2.42	0.55	60.45	-8.09
13	ZINC89137230	Yes	Yes	Yes	Yes	Yes	3.4	0.56	68.27	-8.98
14	ZINC89374817	Yes	Yes	Yes	Yes	Yes	2.34	0.55	109.66	-8.09
15	ZINC78168866	Yes	Yes	Yes	Yes	Yes	2.89	0.85	68.27	-8.28
16	ZINC89374817	Yes	Yes	Yes	Yes	Yes	2.34	0.55	109.66	-7.97
17	ZINC5110352	Yes	Yes	Yes	Yes	Yes	3.3	0.55	127.07	-8.75
18	ZINC89376994	Yes	Yes	Yes	Yes	Yes	1.93	0.55	109.66	-7.88
19	ZINC91165881	Yes	Yes	Yes	Yes	Yes	3.3	0.55	59.57	-8.6
20	ZINC93714769	Yes	Yes	Yes	Yes	Yes	2.38	0.85	64.64	-8.14

SwissDock

The SwissDock is a web software that predicts the interaction between a molecule and a target. Using SwissDock, each of the 20 molecules obtained from ZINCPharmer and the four original molecules selected from the literature were individually docked with the target protein. Following this, binding energies derived from the outcomes were contrasted and tabulated. Myosin is selected as the target protein, identified pharmaceutical compounds and molecules that bind to it. We examined numerous articles and databases about myosin and its function in MMPDS. The search yielded the following four myosin inhibitors: pyridostigmine, rivastigmine, benztropine, and butanedione. These drugs possess a multitude of functions and clinical applications [7, 8].

## Results

ZINC15 was utilized to determine the structure of the four myosin inhibitors that were chosen from the literature (Figure 2). Using SwissADME, the properties of the molecules were evaluated (Table 2). Protein data banks were consulted to acquire the structure of the protein myosin (Figure 3). For the docking process, the 20 novel pharmacophore models that obtained the highest score were eliminated [9]. The molecular docking analysis, conducted utilizing SwissDock, identified two novel molecules (ZINC035924607 and ZINC5110352) that exhibited the most prominent binding patterns (-9.51 and -8.75, respectively), surpassing the binding patterns of the established myosin inhibitors.

A comparison was made between the properties of each molecule and the two novel compounds, which had superior properties that complied with all the established criteria (Table 2). In addition to bioavailability, Log P, and Topological Polar Surface Area (TPSA), additional parameters were compared.

**Table 2:** Properties of existing and novel molecules Log P- Partition coefficient; TPSA- Topological polar surface area Drug likeliness Rules: Lipinski (Rule 1), Ghose (Rule 2), Veber (Rule 3), Egan (Rule 4), and Muegge (Rule 5). Binding energy- Sum of all the intermolecular interactions present between ligand & target.

S. No	Name	Rule1	Rule2	Rule3	Rule4	Rule5	LogP	Bioavailability	TPSA	Binding Energy (kcal/mol)
1	Pyridostigmine	Yes	Yes	Yes	Yes	No	-1.25	0.55	33.42	-7.25
2	Rivastigmine	Yes	Yes	Yes	Yes	Yes	3.2	0.55	32.78	-7.54
3	Butanedione	Yes	No	Yes	Yes	No	1.19	0.55	34.14	-5.86
4	Benztropine	Yes	Yes	Yes	Yes	Yes	3.56	0.55	12.47	-8.16
5	ZINC035924607	Yes	Yes	Yes	Yes	Yes	4.34	0.55	87.72	-9.51
6	ZINC5110352	Yes	Yes	Yes	Yes	Yes	3.3	0.55	127.07	-8.75

**Figure 5:**
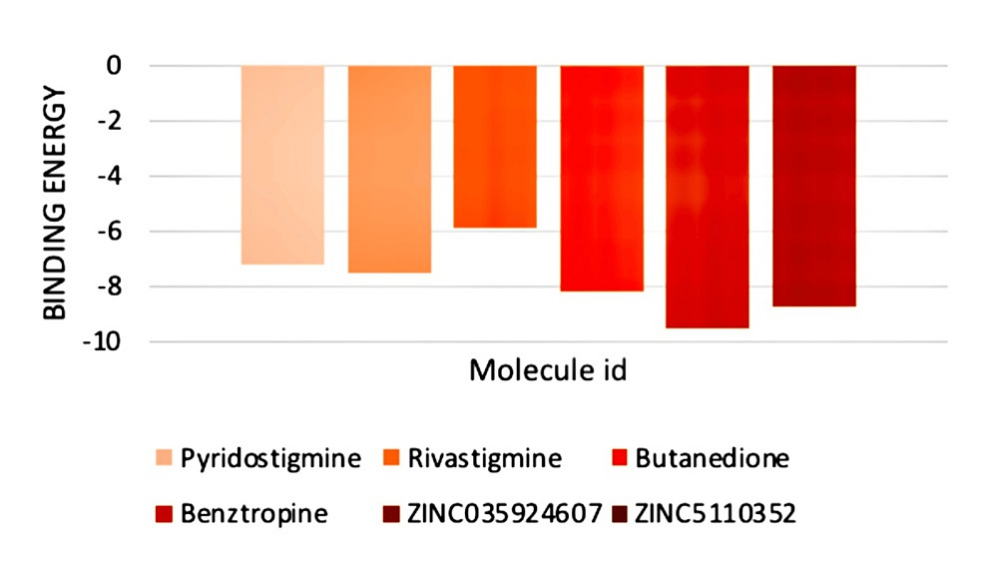
Binding energies of the existing and new molecules

The SwissDock molecular docking analysis discovered two novel compounds (ZINC035924607 and ZINC5110352) with much stronger binding patterns (-9.51 and -8.75) compared to the existing myosin inhibitors (Figure 5). The properties of all the molecules were compared; these two novel compounds demonstrated superior properties by the criteria required for a pharmaceutical substance, including Lipinski (Rule no 1), Ghose (Rule no 2), Veber (Rule no 3), Egan (Rule no 4), and Muegge (Rule no 5) (Table 2) [10]. Additional factors were evaluated in addition to bioavailability, log P, and TPSA [10].

## Discussion

The primary etiology of temporomandibular joint (TMJ) dysfunction is a disturbance in the synchronized functioning of the associated muscles, primarily attributed to a malocclusion. This condition is frequently aggravated by psychosocial variables that contribute to the development of neuromuscular stress. Masticatory myofascial pain dysfunction syndrome (MMPDS) is a condition that involves regional muscle pain, characterized by the presence of localized muscle tenderness and discomfort in tense bands of skeletal muscles [11].

This particular condition is widely recognized as a prevalent etiology of persistent localized pain. A substantial amount of data exists that unequivocally demonstrates the interconnectedness of the mind and body in various facets of pain management, identification, and interpretation. Due to the extended and sometimes diverse treatment stages involved in the management of myofascial pain, healthcare providers must establish a lasting rapport with patients and guide them toward cultivating a favorable mindset toward therapy and a steadfast dedication to long-term transformation [12].

The main goal of molecular docking entailed the anticipation of the interaction between tiny drug-like compounds and target proteins. Numerous diseases arise as a result of protein dysfunction, prompting therapeutic interventions that primarily revolve around the modulation of target proteins through inhibition or activation [13].

The utilization of the free energy concept (δG) is employed in the evaluation of the binding affinity of a protein-ligand complex inside docking studies conducted through the drug discovery tool SwissDock. The observation of a low value and negative value of δG signifies a robust binding affinity between the protein and ligand, suggesting that the ligand adopts a very favorable shape. Pyridostigmine, rivastigmine, butanedione, and benztropine were screened against the target myosin to identify potential pharmaceuticals. The interaction between the drug and ligand was determined by docking ligands against the target.

Potential drugs (pyridostigmine, rivastigmine, butanedione, and benztropine) were screened against the target myosin. Screened ligands were docked against the target to know the interaction between the drug and ligand. Pharmacophore modeling was done for four molecules and validated using the PharmaGist software [14]. A total of 20 lead molecules were selected and screened against myosin protein [15]. The binding energy of new molecules is checked and compared to that of four selected molecules. It is found that among 20 molecules, ZINC035924607 and ZINC5110352 had the highest binding energy and properties superior to that of the molecules chosen and followed all five rules of drug-likeness. This identified molecule may be useful in targeting myosin protein and thus play a major role in drug discovery.

Limitations

The molecular dynamics of the drugs or the resulting molecules were not explored in the present study. Also, the other myosin-related targets and possible drugs must be investigated in future studies.

## Conclusions

The techniques of docking and scoring have undergone substantial advancements in recent years. The utilization of this instrument has proven to be quite advantageous in the process of drug discovery. We conducted a comparative analysis of the prediction capabilities exhibited by each docking and scoring algorithm. The findings of our study indicate that the docking programs examined in this research demonstrate a satisfactory level of performance in docking and are expected to make a substantial contribution to the drug development process. The ligand docking analysis revealed that ZINC035924607 and ZINC5110352 exhibited the highest binding energy among the tested ligands. In summary, our research has yielded a remarkably potent lead chemical that holds promise for the development of a novel, less toxic, and highly effective pharmaceutical agent targeting the masticatory myofascial pain condition.
